# Multisubunit RNA Polymerases of Jumbo Bacteriophages

**DOI:** 10.3390/v12101064

**Published:** 2020-09-23

**Authors:** Maria L. Sokolova, Inna Misovetc, Konstantin V. Severinov

**Affiliations:** 1 Center of Life Sciences, Skolkovo Institute of Science and Technology, 121205 Moscow, Russia; Inna.Misovetc@skoltech.ru; 2 Waksman Institute of Microbiology, Rutgers, The State University of New Jersey, Piscataway, NJ 08854, USA; severik@waksman.rutgers.edu

**Keywords:** jumbo phages, RNA polymerase, multisubunit RNA polymerase, non-canonical RNA polymerases, transcription, promoter, RNA sequencing, pseudo-nucleus, uracil

## Abstract

Prokaryotic viruses with DNA genome longer than 200 kb are collectively referred to as “jumbo phages”. Some representatives of this phylogenetically diverse group encode two DNA-dependent RNA polymerases (RNAPs)—a virion RNAP and a non-virion RNAP. In contrast to most other phage-encoded RNAPs, the jumbo phage RNAPs are multisubunit enzymes related to RNAPs of cellular organisms. Unlike all previously characterized multisubunit enzymes, jumbo phage RNAPs lack the universally conserved alpha subunits required for enzyme assembly. The mechanism of promoter recognition is also different from those used by cellular enzymes. For example, the AR9 phage non-virion RNAP requires uracils in its promoter and is able to initiate promoter-specific transcription from single-stranded DNA. Jumbo phages encoding multisubunit RNAPs likely have a common ancestor allowing making them a separate subgroup within the very diverse group of jumbo phages. In this review, we describe transcriptional strategies used by RNAP-encoding jumbo phages and describe the properties of characterized jumbo phage RNAPs.

## 1. Multisubunit RNA Polymerases of Cellular Organisms

Transcription, the synthesis of RNA from DNA template, is the first step of gene expression. In all cellular organisms from bacteria to humans, transcription of genomic DNA is catalyzed by evolutionarily-related multisubunit RNA polymerases (RNAPs) [1]. In bacteria and archaea, a single enzyme is employed for transcription of all genes, while in eukarya, there are at least three specialized RNAPs dedicated to transcription of different subsets of nuclear genes [1,2]. Archaeal RNAP is remarkably similar to eukaryal RNAP II, which synthesizes messenger RNAs [1,3,4].

**Figure 1:**
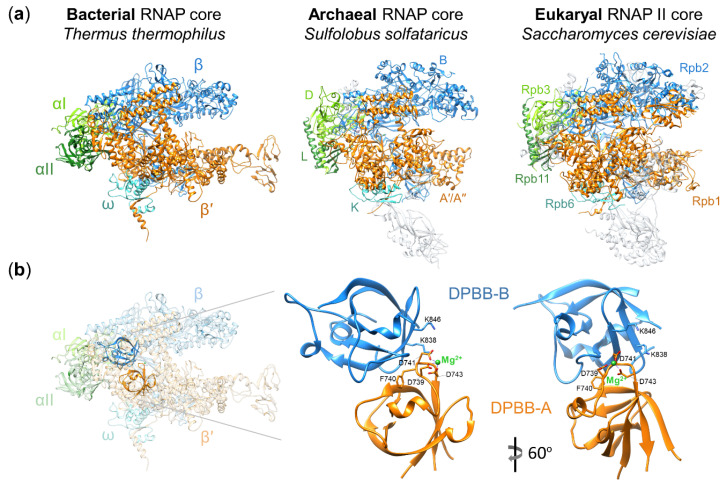
Architecture of multisubunit RNA polymerases (RNAPs). (**a**) Crystal structures of the bacterial RNAP core (PDB: 2O5J) [11], archaeal RNAP core (PDB: 2PMZ) [3], and eukaryal RNAP II core (PDB: 1Y1W) [12] are shown as ribbon diagrams. Homologous subunits are labeled and shown in the same colors in all three structures; archaeal/eukaryal specific subunits are grey and semitransparent; (**b**) left: crystal structure of the bacterial RNAP core (PDB: 2O5J) [11] with elements other than the double-psi β-barrel (DPBB) domains made semitransparent; right: an enlarged view of the two DPBB domains (DPBB-A and DPBB-B from the β′ and β subunits, correspondingly). The active site is located at the interface of the two domains. Some of the universally conserved amino acid residues (aspartates that coordinate the Mg^2+^ ion and lysines that interact with the backbone of the RNA transcript at the −1/−2 positions in the transcribing RNAP [7]) are labeled and shown as stick representation.

Structural and functional studies revealed that the core of multisubunit RNAPs is highly conserved in all three domains of life (Figure 1a) [1,5]. The simplest bacterial RNAP core is composed of two large catalytic subunits β′ and β, a dimer of α subunits (an assembly platform for large subunits), and the ω subunit (a chaperon for β′) (Figure 1a) [6,7,8]. The active site is located at the interface of two double-psi β-barrel (DPBB) domains of β′ and β (Figure 1b). The DPBB domain of the β′ subunit carries the universally conserved amino acid motif DxDGD (where x represents a bulky residue) whose three aspartates coordinate Mg^2+^ ions required for catalysis [9,10]. The archaeal and eukaryal RNAPs core enzymes contain homologs of all bacterial RNAP core subunits as well as multiple additional subunits (Figure 1a) [1,5].

RNAP synthesizes RNA in a template-dependent manner in the course of the transcription cycle, which can be subdivided into three stages: initiation, elongation, and termination of transcription. RNAP core enzyme is catalytically active and can operate alone during the elongation and termination stages but requires accessory factors for promoter-specific transcription initiation. For this purpose, bacteria employ one of several σ factors, each of which binds the RNAP core forming a holoenzyme able to recognize promoters with different consensus elements [13]. Archaeal and eukaryal RNAPs use a complex set of general and specific transcription factors, which are evolutionarily unrelated to bacterial σ factors [14,15]. Most archaeal and eukaryal transcription initiation factors first bind promoter DNA and then recruit the RNAP core [14,15].

During the past two decades, extensive genome sequencing revealed genes coding for distant homologs of cellular RNAPs in the genomes of some eukaryotic viruses, bacteriophages, prophages and likely mobile elements located in the genomes of some *Firmicutes* and *Cyanobacteria*, and in fungal killer plasmids [16,17,18,19,20,21,22]. Some of these genes were shown to encode functional RNAPs [23,24,25,26,27,28], while the products of others remain uncharacterized. These partially characterized and non-characterized putative RNAPs are collectively referred to as “non-canonical RNAPs” since they are highly divergent from multisubunit RNAPs of cellular organisms. While none of these enzymes have been studied to the extent “canonical” enzymes were, investigation of non-canonical RNAPs will be required to achieve a comprehensive view of the function, structure, and evolution, including the evolutionary origins, of multisubunit RNAPs.

## 2. A Historical Expose: The First Multisubunit Phage RNA Polymerase

The story of multisubunit phage RNAPs starts back in the early nineteen-seventies, when it had been shown that infection of *Bacillus subtilis* by phage PBS2 was insensitive to the addition of rifampicin, a drug targeting bacterial RNAP, even if the drug was administered before the infection [29,30]. This result is in contrast to those obtained with *Escherichia coli* T4 and *B. subtilis* SP01 phages, whose development was strongly inhibited by rifampicin because their genes are transcribed by host RNAP [31,32]. The *E. coli* phage T7 was sensitive to rifampicin (and therefore required the host RNAP) only during the first minutes of infection [33]; at later stages, T7 infection was resistant to the drug because the phage utilizes its own single-subunit rifampicin-resistant RNAP [34].

Inspired by the unusual property of PBS2 infection, the group of Richard Losick and Janice Pero purified a rifampicin-resistant RNAP from *B. subtilis* cells infected with PBS2 [35]. The PBS2 RNAP turned out to be a multisubunit enzyme composed, depending on the purification procedure, of either four or five subunits [35,36]. Based on their molecular weights, not a single subunit of the PBS2 RNAP corresponded to the multisubunit RNAP of the host bacterium. Judging from RNA/DNA hybridization experiments, the PBS2 RNAP was specifically transcribing late PBS2 genes in vitro [36]. Despite it being distinct from both multisubunit bacterial RNAP and single-subunit RNAP of phage T7 and its relatives [35,36], the PBS2 RNAP remained largely forgotten and its properties and evolutionary origins stayed unknown for several decades.

## 3. Jumbo Phages Encoding RNA Polymerases and Their Features

At the beginning of the 2000s, several independent studies suggested that jumbo phages related to the *Pseudomonas aeruginosa* phage phiKZ encode two sets of unusual proteins with similarities to different parts of the largest subunits of cellular RNAPs (β′ and β subunits in bacterial RNAP) [37,38,39,40]. The genome of jumbo phage AR9 infecting *B. subtilis*, a close relative of the PBS2 phage, was sequenced and shown to contain genes orthologous to the RNAP genes of phiKZ-like phages [18], suggesting that the PBS2 RNAP purified a long time ago [35,36] in fact belongs to this new group of non-canonical jumbo phage RNAPs. At the time of this writing, several dozens of jumbo phages encoding distant homologs of the β′ and β subunits are known [18,37,38,39,40,41,42,43,44,45,46,47,48,49,50,51,52,53,54,55,56,57,58,59,60,61,62]. They infect diverse bacterial hosts including members of *Pseudomonas*, *Salmonella*, *Yersinia*, *Erwinia*, *Vibrio*, *Ralstonia*, *Bacillus*, *Aeromonas*, *Serratia*, *Klebsiella*, *Escherichia*, and other genera [18,37,38,39,40,41,42,43,44,45,46,47,48,49,50,51,52,53,54,55,56,57,58,59,60,61,62]. Remarkably, hosts of jumbo phages encoding two RNAPs include both Gram-positive and Gram-negative bacteria. It is thus possible that the ancestor of phages of this group could have acquired bacterial RNAP genes before the divergence of Gram-positive and Gram-negative bacteria. In addition to RNAPs, these jumbo phages encode a set of core proteins, including phosphoesterase, DnaB-like replicative helicase, large terminase subunit, the split family B DNA polymerase, the SbcCD complex ATPase, RNA helicase, and ribonuclease H [18]. Jumbo phages encoding their own RNAPs likely rely on a common transcriptional strategy during the infection. Jumbo phages that do not encode their own RNAPs are evolutionarily unrelated to jumbo phages with RNAPs and usually encode clearly recognizable homologs of bacterial σ factors likely interacting with the host RNAP core and directing it to phage promoters [63,64,65,66,67].

While most jumbo phages encoding RNAPs have unmodified DNA genomes, the genomes of some, for example, PBS2 and AR9 infecting *B. subtilis*, vB_BpuM_BpSp infecting *B. pumilus* and phiR1-37 infecting *Yersinia enterocolitica*, contain uracil in place of thymine [18,45,49,68]. Another jumbo phage with a genome in which thymine has been replaced by uracil, is staphylococcal phage S6 [69]. The genome of S6 has not been sequenced yet although it has been suggested that this phage is related to PBS2 [18,69]. To our knowledge, no other viruses except these jumbo phages are known to have DNA genomes with uracils.

Another distinguishing feature of jumbo phages encoding RNAPs (which, however, relates only for jumbo phages with thymine-containing DNA genomes) is that most of them form a unique nucleus-like compartment within the infected cell, which separates phage DNA, located inside, from the host cell cytoplasm [70,71]. This “pseudo-nucleus” is positioned at the center of the cell by phage-encoded tubulin-like PhuZ proteins [71]. The pseudo-nucleus provides defense against bacterial anti-phage systems such as CRISPR-Cas and restriction–modification systems of different types (except the Type III and Type VI CRISPR-Cas systems that target RNA) [62,72]. The absence of pseudo-nucleus in jumbo phages with uracil-containing genomes may reflect the fact that modified DNA itself is sufficient to protect their genomes from host nucleases and defense systems.

In the course of infection, phage and host proteins are partitioned between the pseudo-nucleus and cytoplasm according to their functions. For example, phage 201phi2-1 proteins involved in DNA replication and transcription (specifically, two proteins that constitute the β′ subunit of the non-virion RNAP) were localized inside the pseudo-nucleus [70]. The host DNA topoisomerase I was also found within the pseudo-nucleus [70]. In contrast, host proteins involved in translation and phage proteins involved in nucleotide metabolism are localized in the cytoplasm and are excluded from the pseudo-nucleus [70]. The mechanism of sorting the proteins between pseudo-nucleus and cytoplasm is unknown.

## 4. Transcriptional Strategy of Jumbo Phages Encoding RNA Polymerases

Phage phiKZ replication was shown to be resistant to rifampicin, indicating that similarly to PBS2, its development is fully independent of host transcription machinery [73]. Bacteriophage AR9, a close relative of PBS2, also replicates without the involvement of host RNAP, though rifampicin leads to a decreased yield of phage progeny [74]. The decreased AR9 yield in the presence of rifampicin was explained by the detrimental effect of rifampicin on *B. subtilis* cell integrity [74,75]. Indeed, the AR9 yield was dependent on *B. subtilis* strain used for infection and correlated with a number of cells that survived the treatment with the drug [74].

The effect of rifampicin on infection was also investigated for four jumbo phages infecting *Ralstonia solanacearum*-phiRP12, phiRP31, RSL2, and RSF1 [41,53]. Interestingly, despite the presence of all jumbo phage RNAP genes in each genome, only phiRP12 and phiRP31 produced phage progeny in the presence of rifampicin, while the multiplication of RSL2 and RSF1 was abolished [41,53]. In comparison to RSL2 and RSF1, the phiRP12 and phiRP31 genomes possess extra regions coding for proteins with unknown functions [53]. It might be that these proteins are somehow associated with lesser dependence of these phages on cell resources compromised by rifampicin. Yet, it cannot be excluded that host RNAPs transcribe some genes of AR9, RSL2, and RSF1 required for efficient infection.

**Figure 2:**
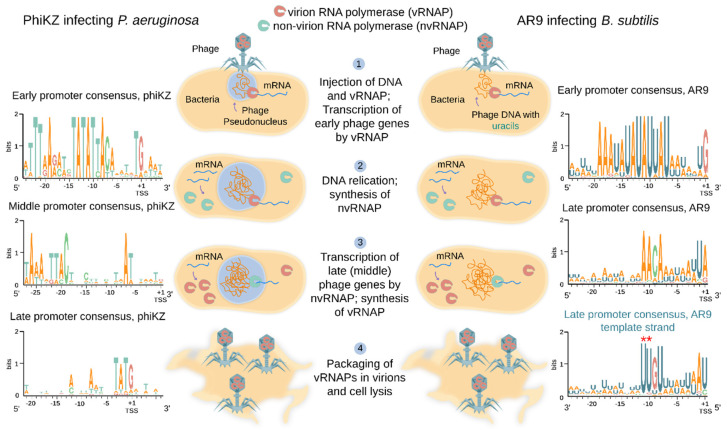
Transcription strategies of jumbo phages encoding two RNAPs. PhiKZ (**left**) and AR9 (**right**) phages are, respectively, representatives of thymine-containing DNA genome phages encoding a pseudo-nucleus, and uracil-containing DNA genome phages that do not encode a pseudo-nucleus. The principal steps of the infection cycle are listed in the middle of the figure. WebLogos of phiKZ early, middle, and late promoters are shown on the left. WebLogos of AR9 early and late promoters are shown on the right. Experimentally identified transcription start sites (TSSs) are marked. For the AR9 late promoter, a WebLogo of the template strand is shown in the right bottom corner. The two uracils crucial for promoter recognition are marked by red asterisks (see explanation in Section 6).

Global analysis of gene expression in cells infected with several RNAP-encoding jumbo phages was performed. As is typical for most lytic phages, the phiKZ genes are expressed in three temporal classes: early, middle, and late [73]. Transcription profiling of *B. subtilis* cells infected with AR9 revealed early, late and continuously expressed classes of genes [74]. AR9 genes from the unusual latter class are transcribed throughout the infection and have both early and late promoters upstream of them [74]. PhiR1-37, another jumbo phage with a uracil-containing DNA genome, also has a large group of continuously expressed genes [76]. Transcription of early phiKZ and AR9 genes was shown to be independent of protein synthesis in the infected cells [73,74]. Together with the independence of these genes’ transcription from host RNAP, this observation points out that the early genes of AR9 and phiKZ are transcribed by the virion-packaged RNAP that must be injected into the host cell along with phage DNA (Figure 2) [73,74]. Indeed whenever it has been investigated, one of the two sets of RNAP β/β′ homologs of jumbo phages is found in virions and thus should constitute the virion RNAP (vRNAP) [18,41,45,77,78]. Transcription of middle and late phiKZ genes, and late genes of the AR9 phage, requires protein synthesis and thus must be performed by an enzyme that is fully or partially synthesized *de novo* after infection [73,74]. In accordance with this expectation, the second set of RNAP β/β′ homologs is encoded by early phiKZ and AR9 genes [73,74] and must therefore form their non-virion RNAP (nvRNAP) synthesized during the infection (Figure 2). Overall, it follows from these observations that RNAP-encoding jumbo phages rely on vRNAP (present in virions) for transcription of early genes and nvRNAP (synthesized during the infection) for transcription of late (and, possibly, middle) genes (Figure 2).

In each jumbo phage encoding RNAPs, a conserved motif can be located bioinformatically upstream of presumed early operons [18]. For all phages for which transcript profiling was performed, the predicted motif was indeed shown to be an early promoter consensus [73,74,76]. While different from phage to phage, these motifs are characterized by ~10-nucleotide AT-rich highly conserved sequences centered approximately at position −10 with respect to the transcription start site (TSS) of early genes (Figure 2) [18,73,74]. They must define early phage promoters recognized by vRNAPs.

Bioinformatics analysis fails to predict middle or late promoters of RNAP-encoding jumbo phages. The 5′ ends of middle and late phiKZ transcripts were identified from RNA sequencing and primer extension experiments [73]. The only apparent commonality found in DNA located upstream of likely TSSs of middle promoters was a weak AT-rich motif centered at position −24 with respect to TSS (Figure 2) [73]. For late promoters, no sequence conservation upstream of 5′ ends of late transcripts could be detected apart from a 5′-T^−3^ATG^+1^-3′ motif overlapping the TSS (Figure 2) [73]. Differential RNA sequencing allowed researchers to determine a consensus motif 5′-A^−11^ACA-(6N)-UA/G^+1^-3′ upstream of late AR9 promoters (Figure 2) [74]. This motif is distinct from either the middle or late promoter consensus motifs of phiKZ.

## 5. Analysis of RNA Polymerases Genes of Jumbo Phages and Their Origins

**Figure 3:**
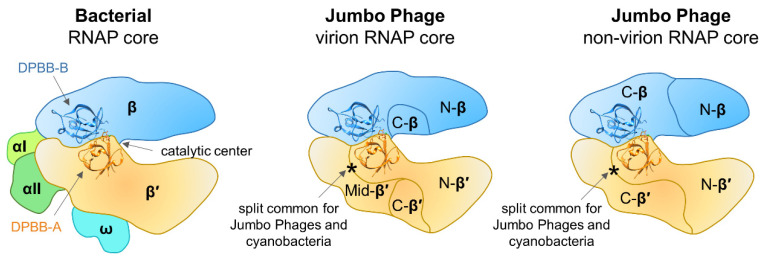
Schematic representation of bacterial and jumbo phage RNAP core enzymes. Structures of DPBB domains are taken from the *T. thermophilus* RNAP crystal structure (PDB: 2O5J) [11]. Jumbo phage RNAPs do not contain homologs of bacterial RNAP α and ω subunits. The virion RNAP (vRNAP) core is composed of at least five polypeptides: N-β′, Mid-β′, C-β′, and N-β, C-β, homologous to the N-terminal, middle, and C-terminal parts of bacterial β′, and N-terminal and C-terminal parts of bacterial β, correspondingly. The non-virion RNAP (nvRNAP) core is composed of four polypeptides: N-β′, C-β′, and N-β, C-β, homologous to the N-terminal and C-terminal parts of β′, and N-terminal and C-terminal parts of β, correspondingly. The DPBB-A is present in the N-terminal parts of β′ in both vRNAP and nvRNAP. The split site after DPBB-A (marked by asterisk) is common for vRNAP, nvRNAP, and cyanobacterial RNAP.

Sequence and structural features of multisubunit RNAPs in all three domains of life were described in detail by Lane and Darst [7,79]. The sequences of the jumbo phage β′/β-like subunits were compared with those of corresponding subunits of *Thermus thermophilus* RNAP and most of the universally conserved RNAP regions were identified including the two DPBB domains and the amino acid motifs that comprise the catalytic center [18,28]. Initially, the protein corresponding to the very end of the β′ subunit of vRNAP was not found in the genomes of jumbo phages. However, subsequent investigation of essential genes of *Salmonella* jumbo phage SPN3US identified a small virion protein gp244 that corresponds to the C-terminal-most bacterial RNAP β′ conserved region [80]. Homologs of SPN3US gp244 can be identified in all RNAP-encoding jumbo phages [80]. Thus, the core of the jumbo phage vRNAP should contain at least five polypeptides that together correspond to full-length β′/β subunits of bacterial RNAP, while the core of nvRNAP contains at least four polypeptides corresponding to full-length β′/β (Figure 3).

Virion RNAPs of *Bacillus* jumbo phages (AR9, PBS1, vB_BpuM_BpSp) have a unique N-terminal HD nuclease domain fused to subunits corresponding to C-terminal fragment of bacterial RNAPs β subunits [18,49]. This domain is present in enzymes that are either known or predicted to possess phosphohydrolase activity and appear to be involved in nucleic acid metabolism or signal transduction in bacteria, archaea, and eukaryotes [81]. The role that the HD domain plays in vRNAP function and phage transcription is unknown.

With available data, it is impossible to trace whether the vRNAP and nvRNAP emerged as a result of gene duplication in the ancestral jumbo phage genome or there were two independent acquisitions of RNAP genes from the host. It was proposed that split jumbo phage RNAP genes could have appeared due to enhanced intron mobility in the ancestral phage followed by the deletion of the introns and genome rearrangements [18]. However, the β′-like subunits of vRNAP and nvRNAP have a common split-site (following the DPBB domain), whose position coincides with a split found in cyanobacterial homologs (Figure 3) [82] but not with that found in archaeal RNAPs [4]. Based on the location of split sites in β′-like subunits of both vRNAP and nvRNAP, it can be hypothesized that these RNAPs were acquired from cyanobacteria, though the alternative hypothesis of their independent appearance can not be excluded either. The jumbo phages β-like subunits of vRNAP and nvRNAP have split sites in different positions (Figure 3) that do not match that found in archaea [4]. Thus, these splits must have appeared independently in the ancestral jumbo phage and were not inherited from a cellular ancestor.

The uniquely organized multisubunit jumbo phage RNAPs should show notable differences from their cellular counterparts in terms of function. First, they lack compulsory components of multisubunit RNAP core enzymes (α, ω) (Figure 3). Second, jumbo phage genomes do not encode recognizable homologs of any known transcription initiation factors. Thus, it is likely that RNAPs of jumbo phages have unique properties: they must rely on an alternative mechanism of assembly of the core complex and may utilize novel transcription initiation strategies; other aspects of transcription may also significantly differ.

## 6. In Vitro Properties of Non-Virion RNA Polymerases of Jumbo Phages

Though no vRNAP has been purified to date, nvRNAPs encoded by PBS2, phiKZ, and AR9 phages have been obtained and characterized to different extents [26,28,35,36]. The phiKZ nvRNAP was shown to consist of four predicted phage proteins jointly comprising the full-length β’- and β-like subunits and a fifth subunit gp68 with no sequence similarity to functionally characterized proteins [28]. Homologs of phiKZ gp68 are found in all other jumbo phage genomes encoding β’- and β-like subunits. The phiKZ nvRNAP did not transcribe from DNA fragments containing middle phiKZ promoters in vitro. It was suggested that an alternative form of phiKZ nvRNAP may exist and transcribe from the middle promoters [28]. In contrast, phiKZ nvRNAP efficiently transcribes in vitro from DNA fragments containing phiKZ late promoters containing the 5′-T^−3^ATG^+1^-3′ consensus element [28]. All four conserved nucleotides are strictly required for transcription initiation [28]. Since neighboring nucleotides can be substituted without affecting the in vitro function, it is not clear what determines the specificity of late promoter recognition since the short consensus motif frequently present in the genome can not be sufficient for promoter-specific transcription on its own. It was shown that the region downstream of TSS is important but no conserved motifs were identified there [28]. Since the phiKZ phage as many other jumbo phages with RNAPs and thymine-containing genomes forms a pseudo-nucleus shielding its DNA during infection [62,70,71,72], it is possible that phage DNA has a specific arrangement within the compartment that affects promoter recognition by phiKZ nvRNAP in vivo.

Similarly to the phiKZ nvRNAP, the AR9 nvRNAP consists of β’ and β homologs and the fifth protein gp226 (a homolog of phiKZ gp68) [26]. In contrast to phiKZ nvRNAP it was possible to purify the four-subunit AR9 nvRNAP core complex composed of β’ and β homologs only [26,83]. The AR9 nvRNAP is catalytically active but cannot initiate transcription from promoters [26]. The initiation-competent AR9 nvRNAP holoenzyme contains the fifth protein, gp226, which is thus formally equivalent to bacterial RNAP σ factors despite the lack of sequence similarity. The holoenzyme recognizes late phage promoters with a template strand sequence 3′-U^−11^UGU-(6N)-AU/C^+1^-5′ (Figure 2) [26]. The same sequence containing thymines instead of uracils is not recognized and uracils at positions −11 and −10 are strictly required for promoter recognition [26]. It was proposed that the requirement for uracils in promoter consensus element is a strategy that should allow AR9 (and perhaps other jumbo phages with uracil-containing DNA genomes) to avoid unnecessary transcription from host DNA, which contains multiple matches to the simple consensus of phage late promoter [26].

While the requirement for an additional core-binding factor to initiate transcription is similar to that of bacterial RNAPs, in contrast to the σ^70^-RNAP holoenzymes which bind sequence specifically to the non-template strand of their −10 promoter element (T^−12^ATAAT^−7^) [26,84,85] the AR9 nvRNAP holoenzyme recognizes the template strand of its promoter (3′-U^−11^UGU-(6N)-AU/C^+1^-5′) (Figure 2) and is even capable of efficient promoter-specific transcription from single-stranded DNA [26]. The molecular mechanism of non-template strand recognition by σ^70^-RNAP holoenzymes is known: the A^−11^ and T^−7^ bases of the −10 element are flipped and bound by the σ2 domain of σ factors in RNAP holoenzymes [85,86] (C^−10^ of the consensus sequence G^−12^TC^−10^ plays this role for σ^E^-RNAP holoenzyme [87]). The U^−11^ and U^−10^ of the template strand of AR9 late promoters may be specifically recognized by AR9 nvRNAP in a similar way, through base flipping and burying into protein pockets. In support of this hypothesis, it was shown that U^−11^ and U^−10^ bases become protected from modification by KMnO_4_ in AR9 nvRNAP-promoter complexes formed on single-stranded DNA, indicating their tight interaction with the enzyme [83]. The low sensitivity to KMnO_4_ treatment was previously observed for single-stranded T^−7^ of the –10 promoter element in the open promoter complex formed by the σ^70^-RNAP holoenzyme [88]. The fact that AR9 nvRNAP does not bind and does not melt thymine-only late promoter templates suggests that 5-methyl groups of thymines at positions −11 and −10 of the template strand interfere with the recognition by AR9 nvRNAP [26].

Although we do not know yet the biological role of promoter-specific transcription of single-stranded DNA by AR9 nvRNAP, the ability of AR9 nvRNAP to transcribe single-stranded DNA implies that its elongation and termination properties are different from those of canonical multisubunit RNAPs. The non-template DNA strand was shown to play a crucial role in RNA displacement from the RNA-DNA hybrid during transcription by cellular multisubunit RNAPs [89,90]. If AR9 nvRNAP also separates RNA from single-stranded DNA during transcription elongation, it must accomplish this by a unique mechanism that is absent in cellular enzymes. It was shown that in in vitro single-stranded DNA transcription reactions, a significant amount of AR9 nvRNAP-synthesized transcripts are present in a free form when the concentration of DNA template is lower than that of RNAP, indicating the ability of the enzyme to separate RNA from single-stranded DNA, i.e., in the absence of non-template strand [83]. The RNA transcript may be separated from the single-stranded DNA template in two ways. The first way is the direct displacement of the nascent RNA from the RNA-DNA hybrid achieved through an unknown structural feature that should distinguish the AR9 nvRNAP from other cellular RNAPs. Alternatively, it is possible that the first round of transcription by AR9 nvRNAP leads to the formation of RNA-DNA fork-junctions with nascent RNA occupying the position of non-template DNA strand downstream of TSS. This annealed RNA can be displaced from the hybrid not during its synthesis but in the course of a subsequent round of transcription. This scenario is only possible because AR9 nvRNAP recognizes the template strand of its promoter: it would be impossible for other known RNAPs requiring the non-template promoter DNA strand. The AR9 DNA is very AU-rich (72.25%) [18] and thus it was proposed that phage DNA may be present in a partially single-stranded form in infected cells, especially during phage DNA replication and the unique properties of phage nvRNAP may allow it to utilize such partially single-stranded DNA for specific transcription of late genes [26].

The AR9 nvRNAP core is unable to bind promoter DNA but is catalytically active [26]. How the β’- and β-like subunits of the AR9 nvRNAP (and nvRNAPs of other jumbo phages) are kept together in the absence of additional subunits (especially considering that both counterparts of the β’ and β are split into several polypeptides) remains unknown. The arrangement where catalytic subunits of a multisubunit RNAP alone constitute an active RNAP core in the absence of additional subunits is a unique one. The α subunits forming the assembly platform likely were present in the multisubunit RNAP of the Last Universal Common Ancestor since all cellular multisubunit RNAPs contain them (Figure 1a). The absence of the assembly platform from jumbo phage nvRNAP core (and, likely, from vRNAPs) likely means that RNAPs of jumbo phages derived from cellular enzymes must have evolved in a way that strengthened the interactions between the catalytic subunits which allowed the loss of the assembly platform.

## 7. Concluding Remarks

In this contribution, we described transcriptional strategies used by RNAP-encoding jumbo phages and properties of jumbo phage nvRNAPs characterized to date. The vRNAP has not been yet purified from any jumbo phage. In addition to potentially unique transcription properties, the vRNAPs may possess remarkable folding capabilities because they are likely delivered into the cell through a narrow tail channel in an unfolded state and their subunits must efficiently find each other upon the delivery into the host. These properties, if confirmed, can be of interest for biotechnological applications.

The investigation of jumbo phage RNAPs is important for the understanding of multisubunit RNAPs evolution and tracing the possibly ancient origins of RNAP-encoding jumbo phages. Recently, a distinct group of multisubunit RNAP-encoding phages was discovered by analyzing metagenomics data [91]. Thorough phylogenetic analyses placed the newly identified RNAPs as a separate branch on the phylogenetic tree and these RNAPs were proposed to be acquired from an ancient cell before the split of eubacteria from other kingdoms [91]. The authors excluded the jumbo phage RNAPs from their analysis because it was impossible to confidently place them on the phylogenetic tree likely due to the Long Branch Attraction effect caused by their rapid evolution [91]. Thus, the origins of jumbo phage RNAPs remain a mystery. It is possible, however, that once available sequence information is supplemented with jumbo phage RNAPs structures, building a phylogenetic tree, which includes jumbo phage RNAPs, will become possible. This structural work is currently ongoing in our laboratories.
